# Supplementary material for: Distinct echinocandin responses of Candida albicans and Candida auris cell walls revealed by solid-state NMR
Source: Nat Commun. 2025 Jul 8;16:6295. doi: 10.1038/s41467-025-61678-1 (PMC12238410; doi:10.1038/s41467-025-61678-1)
Supplement: Supplementary file 1 — Supplementary Information [file 41467_2025_61678_MOESM1_ESM.pdf]

Supplementary Information

Distinct Echinocandin Responses of *Candida albicans* and *Candida auris* Cell Walls Revealed by Solid-State NMR

Malitha C. Dickwella Widanage[1,#,$], Kalpana Singh[1,#], Jizhou Li[2, 3,#], Jayasubba Reddy Yarava[1], Faith J. Scott[4], Yifan Xu[1], Neil A.R. Gow[5], Frederic Mentink-Vigier[4], Ping Wang[6], Frederic Lamoth[2, 3*], and Tuo Wang[1*]

[1] Department of Chemistry, Michigan State University, East Lansing, MI, USA

[2] Institute of Microbiology and Service of Infectious Diseases, Lausanne University Hospital and University of Lausanne, Lausanne, Switzerland

[3] Infectious Diseases Service, Lausanne University Hospital and University of Lausanne, Lausanne, Switzerland.

[4] National High Magnetic Field Laboratory, Florida State University, Tallahassee, FL, USA

[5] Medical Research Council Centre for Medical Mycology at the University of Exeter, University of Exeter, Geoffrey Pope Building, Stocker Road, Exeter, EX4 4QD, UK

[6] Departments of Microbiology, Immunology and Parasitology, Louisiana State University Health Sciences Center, New Orleans, LA, USA

[#] These authors contributed equally

[$] Current address: National Renewable Energy Laboratory, Golden, CO, USA

[*] Correspondence: frederic.lamoth@chuv.ch; wangtuo1@msu.edu

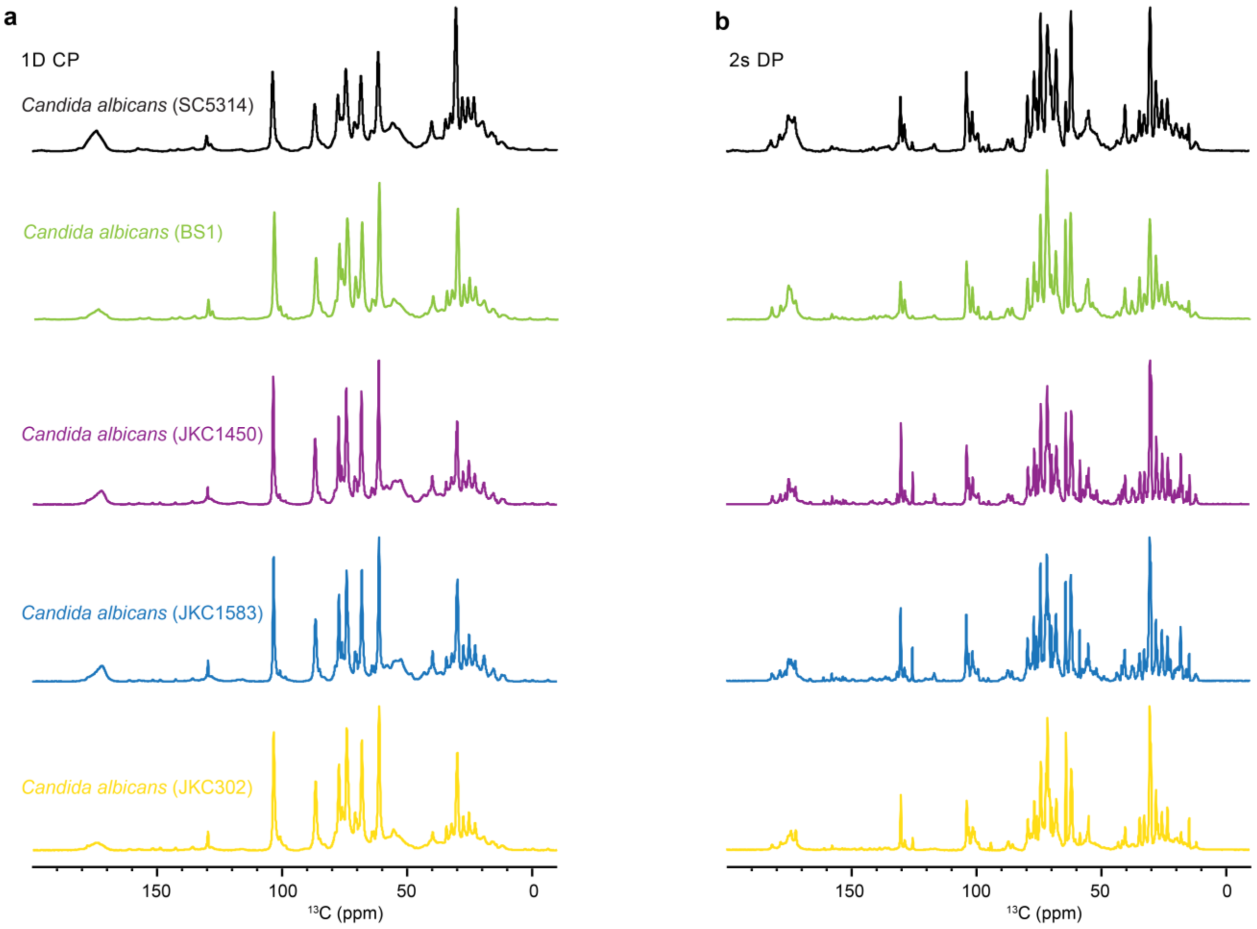

Supplementary Figure 1. 1D ^{13}C spectra of different strains of *C. albicans.* (**a**) 1D ^{13}C CP spectra detecting rigid molecules in five strains, from top to bottom: SC5314, BS1, JKC1450, JKC1583 and JKC302. (**b)** 1D ^{13}C DP spectra with 2 s recycle delays detecting mobile molecules in the same strains. All five strains exhibit highly similar spectral patterns for both the rigid and mobile molecular phases.

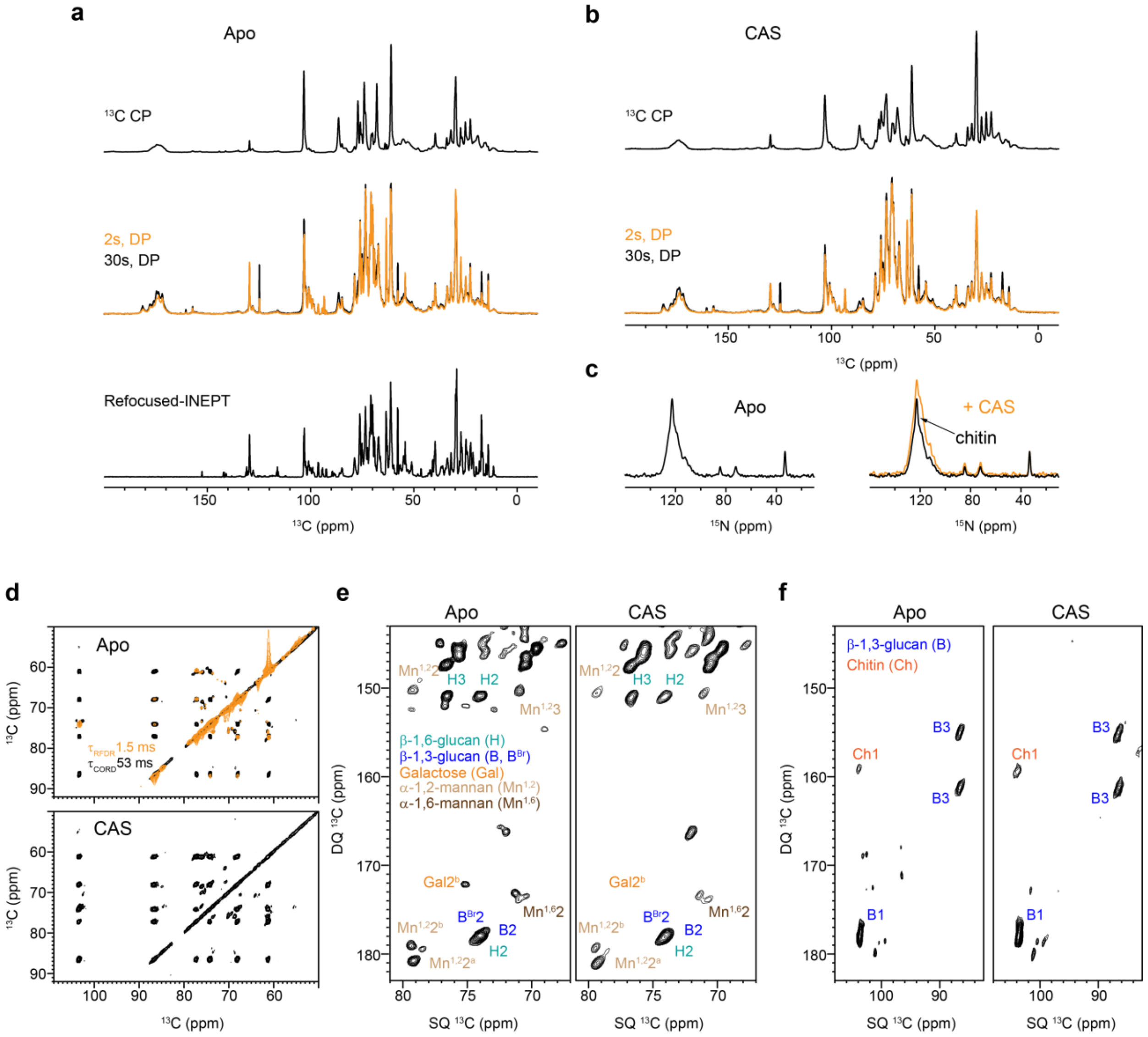

Supplementary Figure 2. Echinocandin-resistance I.3 strain treated with caspofungin at MIC. (**a**) 1D ^{13}C CP (top), ^{13}C DP (middle), and refocused INEPT (bottom) spectra of apo cell wall. (**b**) 1D ^{13}C CP (top) and ^{13}C DP (bottom) spectra of drug-treated cell wall. (**c**) Comparison of 1D ^{15}N CP spectra of cell walls with and without drug. (**d**) Comparison of 2D ^{13}C-^{13}C 53 ms CORD spectra of cell walls without (top) and with drug (bottom). 2D ^{13}C-^{13}C 1.5 ms RFDR spectrum overlay on CORD spectrum for apo cell wall. (**e**) 2D ^{13}C-^{13}C refocused DP-J INADEQUATE spectra of AR387 strain with and without drug cell walls. (**f**) 2D ^{13}C-^{13}C CP INADEQUATE spectra of AR387 strain with and without drug detected rigid cell wall polysaccharides.

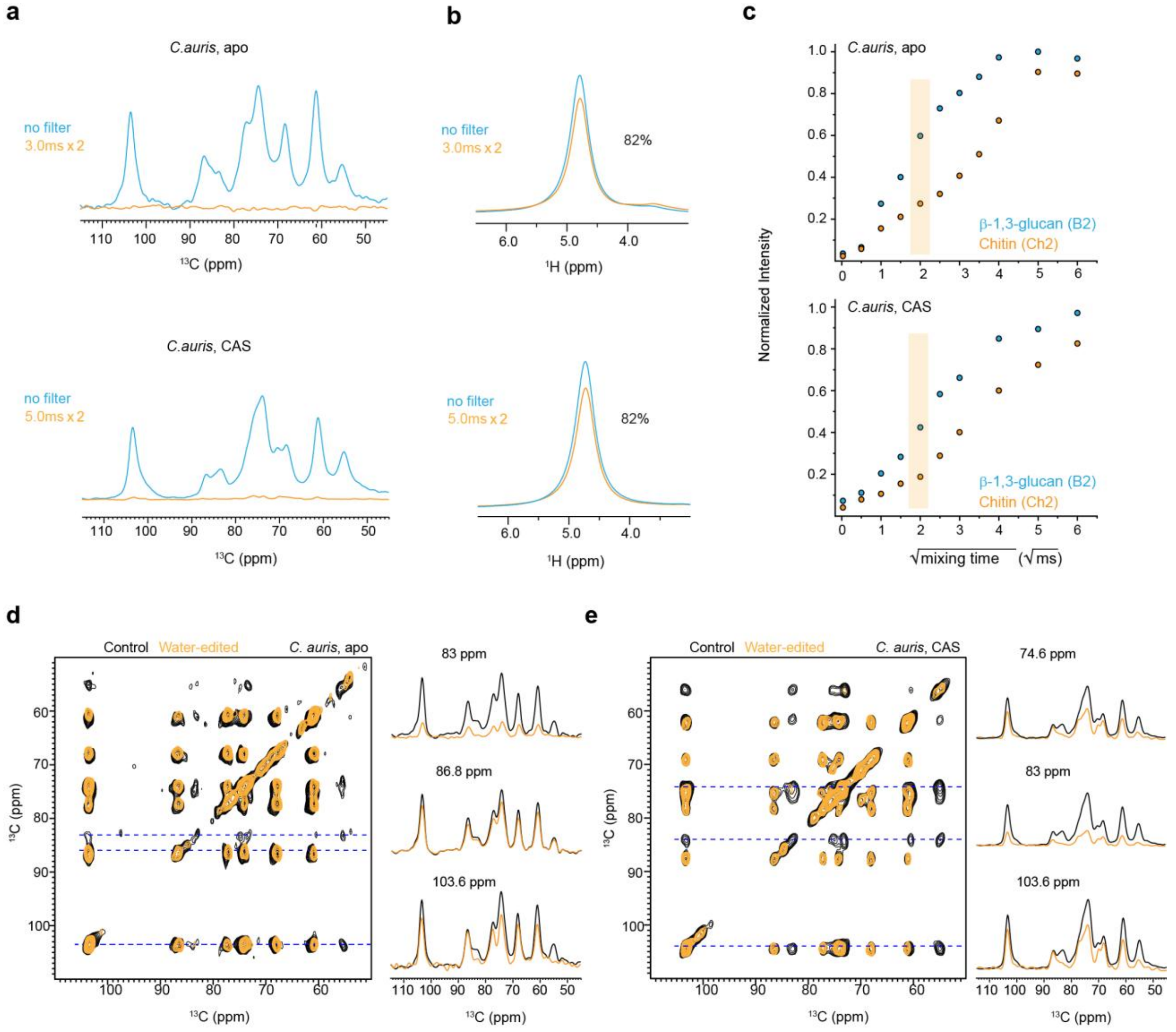

Supplementary Figure 3. Water-edited spectra of *C. auris* to access polymer hydration. (**a**) ^{1}H-T_2 filtered (orange) and control (blue) ^{13}C spectra are shown for apo (top) and caspofungin-treated (bottom) *C. auris* samples. No spin diffusion was applied. Approximately 92% of carbohydrate ^{13}C signals were removed by the ^{1}H-T_2 filter. (**b**) ^{1}H-T_2 filtered (orange) and control (blue) ^{1}H NMR spectra, with 82% of water signal retained for both samples after the ^{1}H-T_2 filter. (**c**) Representative water-to-polysaccharide ^{1}H spin diffusion buildup curves. Overlay of 2D water-edited (orange) and control (black) ^{13}C-^{13}C correlation spectra of (**d**) apo sample and (**e**) caspofungin-treated *C. auris*. Representative 1D slices extracted from the 2D ^{13}C-^{13}C correlation spectra are shown for each sample. The control data are displayed as black solid lines, and the water-edited spectra are plotted in orange. All spectra were measured on a 400 MHz spectrometer at 10 kHz MAS. Source data are provided as a Source Data file.

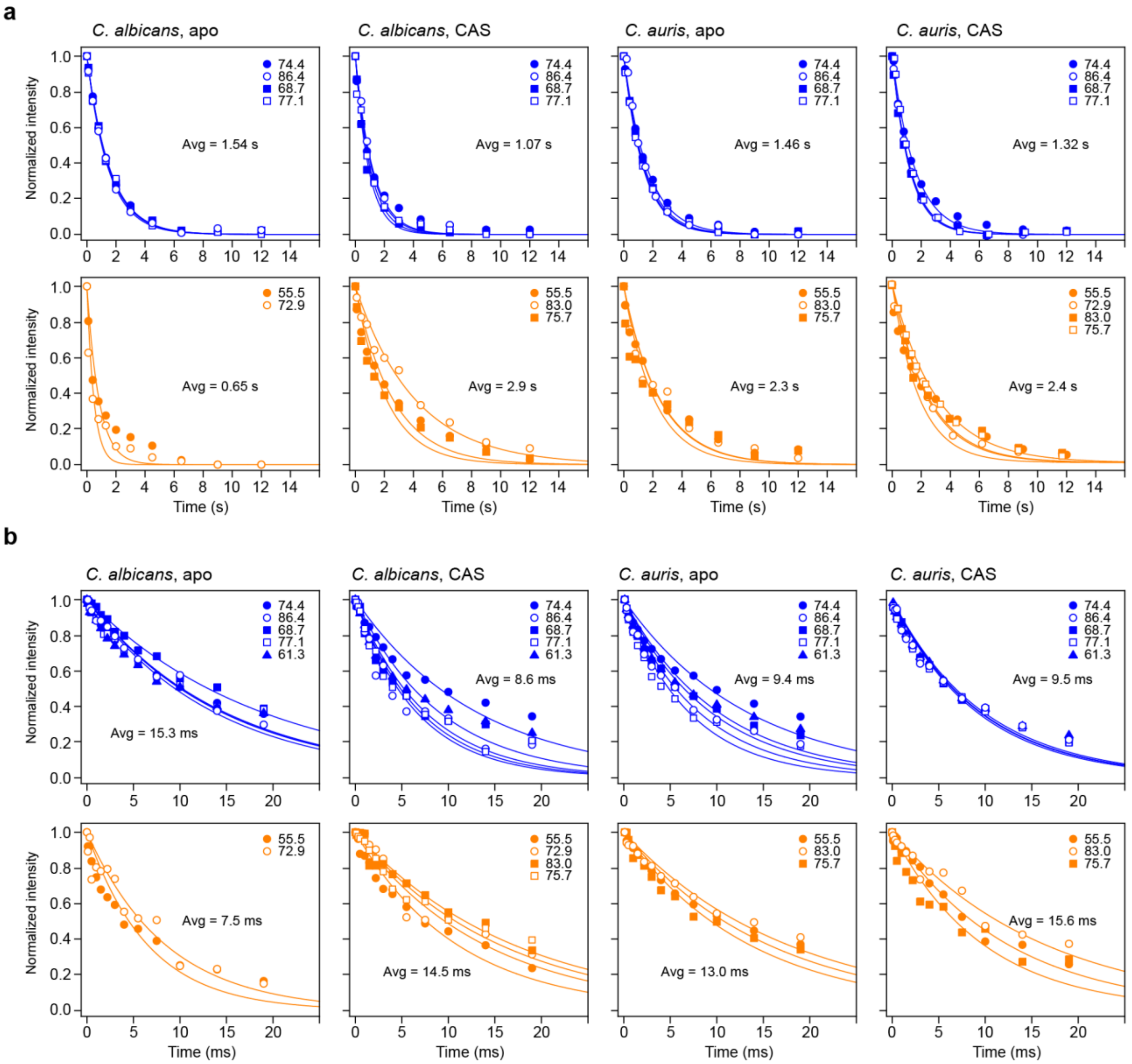

Supplementary Figure 4. Relaxation curves of polysaccharides in *Candida* cell walls. The relaxation decay curves are shown separately for (**a**) ^{13}C T_1 and (**b**) ^{1}H $T_{1\rho}$ of the *Candida* species (SC5314 and AR386) with and without drug-treated cell walls. Source data are provided as a Source Data file.

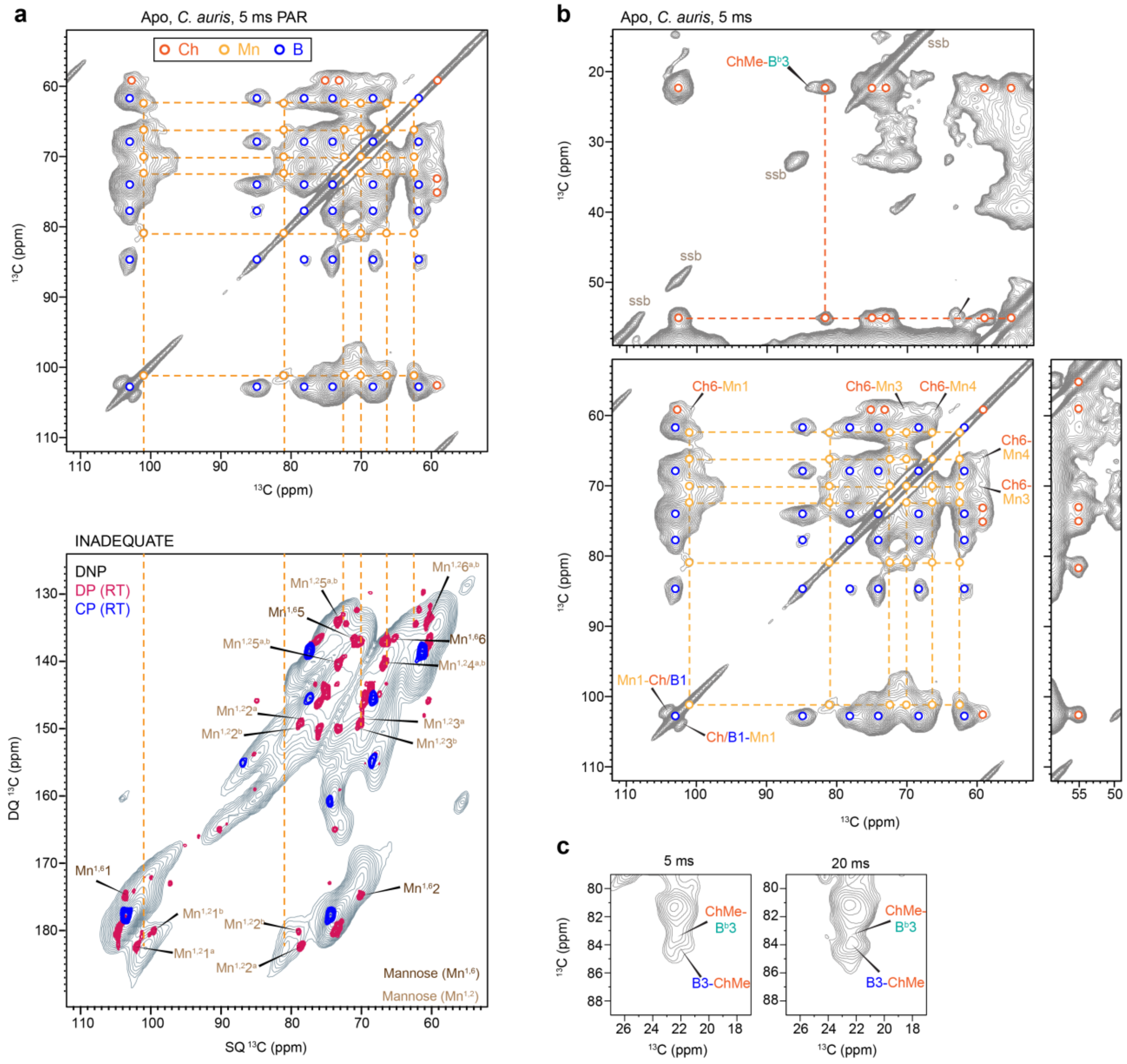

Supplementary Figure 5. Intermolecular interactions of apo *C. auris* cell walls. (**a**) DNP 2D ^{13}C correlation spectra measured with 5 ms PAR (top) pulse sequences on apo *C. auri*s AR386. Intramolecular cross peaks within each molecule are shown using open circles for chitin (orange), β-1,3-glucan (blue) and mannan (orange). For comparison, refocused J-INADEQUATE spectra of *C. albicans* measured under DNP condition (grey) is shown as the bottom panel, with comparison to room-temperature spectra of *C. albicans* measured with CP (blue) and DP (magenta). Signals of α-1,2-linked mannose residues aligned well with the third-type of signals identified in 5 ms PAR in addition to chitin and β-1,3-glucan. (**b**) Intermolecular cross peaks identified in DNP 5 ms PAR spectrum of *C. auris*. (**c**) Zoomed-in region of 5 ms and 20 ms PAR spectra of *C. auris* showing interactions between chitin methyl and β-1,3-glucan carbon 3.

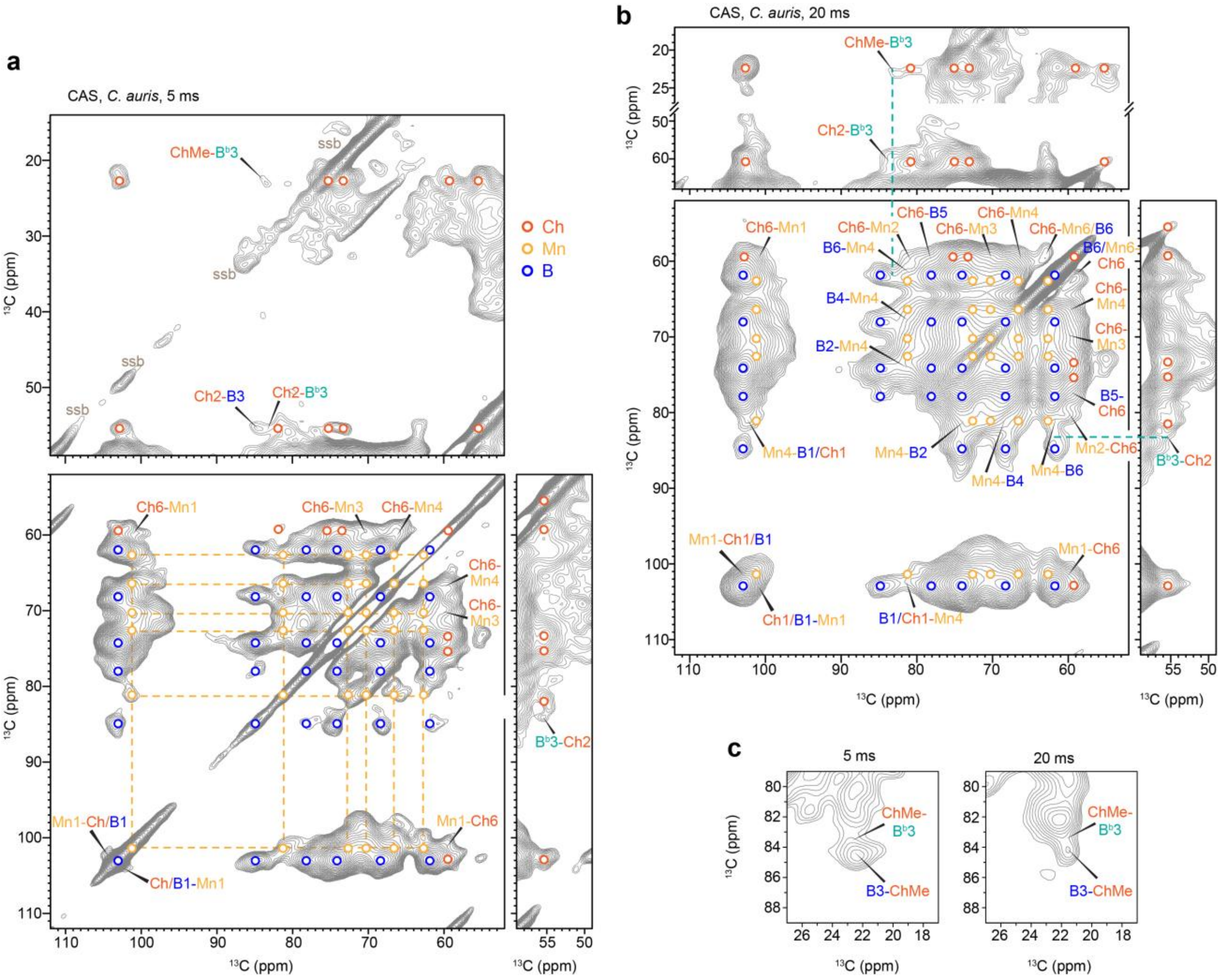

Supplementary Figure 6. Intermolecular interactions of caspofungin-treated *C. auris* cell walls. (**a**) DNP-enhanced 5 ms PAR spectrum of caspofungin-treated *C. auris* sample. Intramolecular cross peaks within each molecule are shown using open circles for chitin (orange), β-1,3-glucan (blue), and mannan (orange). (**b**) Intermolecular cross peaks identified in DNP 5 ms PAR spectrum. (**c**) Zoomed-in region of 5 ms and 20 ms PAR spectra showing interactions between chitin methyl and β-1,3-glucan carbon 3.

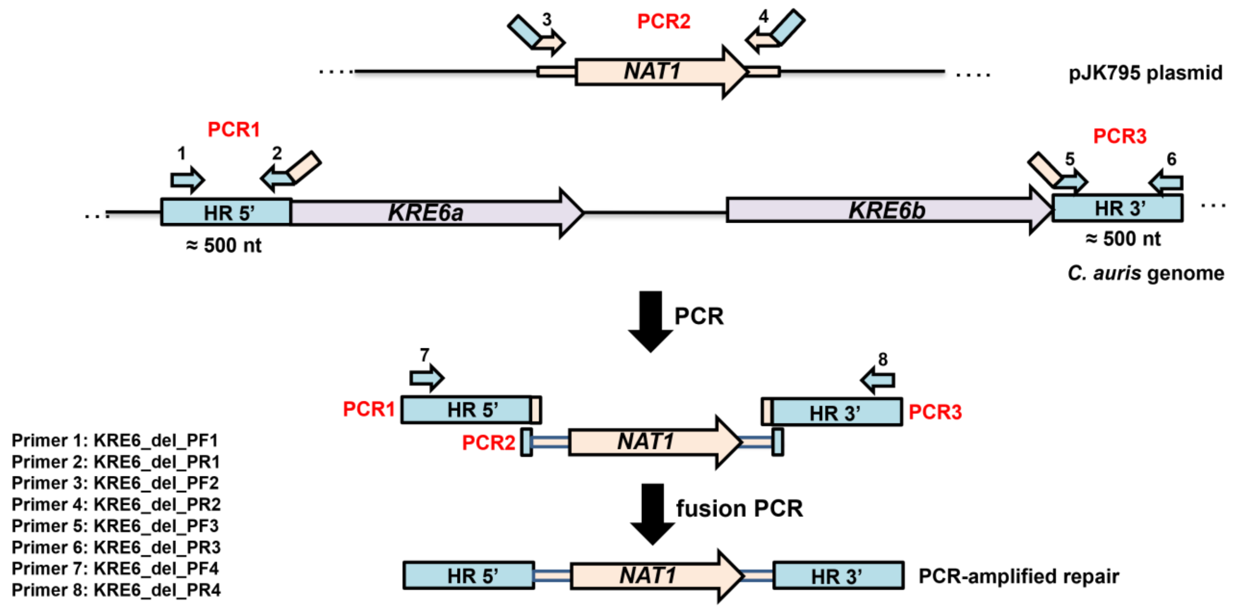

Supplementary Figure 7. Construction of the *C. auris kre6ab*Δ strain. Schematic view of designed fusion PCRs to obtain a PCR-amplified repair fragment containing *NAT1* and the two-sided homolog regions (HR) with about 500 bp in the upstream region of *KRE6a* and downstream region of *KRE6b*. The fusion PCRs were carried out with overlapping primers, as indicated. Primer 2 is reverse complemented to primer 3, and primer 4 is reverse complemented to primer 5.

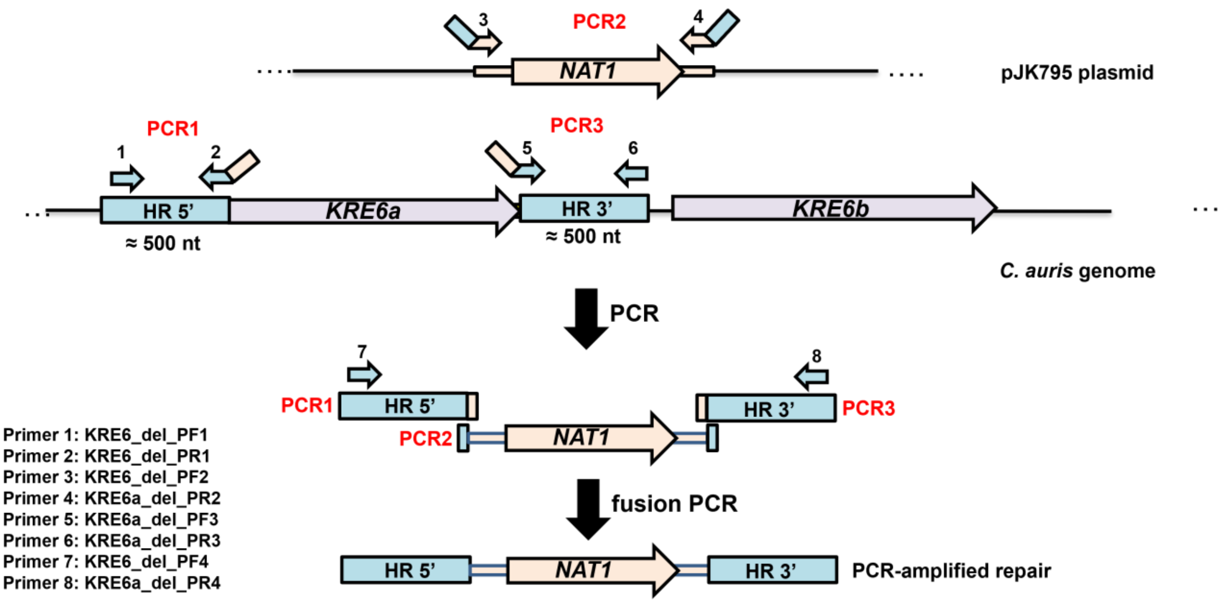

Supplementary Figure 8. Construction of the *C. auris kre6a*Δ strain. Schematic view of designed fusion PCRs to obtain a PCR-amplified repair fragment to delete *KRE6a*. The fragment contains *NAT1* and the two-sided homolog regions (HR) with about 500 bp in the upstream and downstream regions of *KRE6a*. The fusion PCRs were carried out with overlapping primers, as indicated. Primer 2 is reverse complemented to primer 3, and primer 4 is reverse complemented to primer 5.

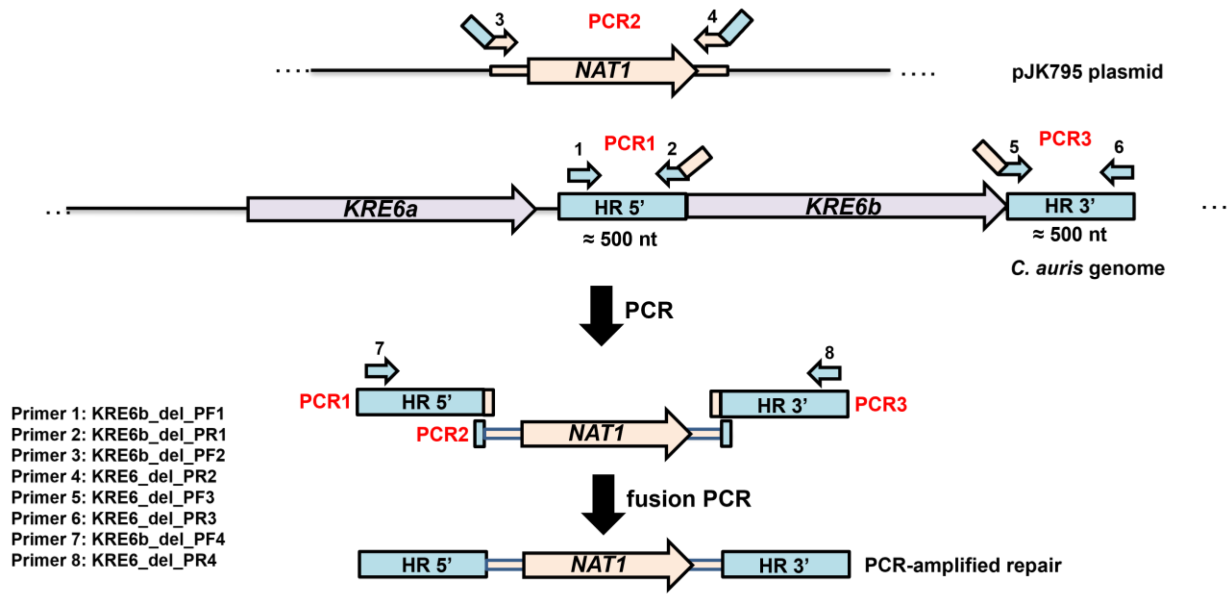

Supplementary Figure 9. Construction of the *C. auris kre6b*Δ strain. Schematic view of designed fusion PCRs to obtain a PCR-amplified repair fragment to delete *KRE6b*. The fragment contains *NAT1* and the two-sided homolog regions (HR) with about 500 bp in the upstream and downstream regions of *KRE6b*. The fusion PCRs were carried out with overlapping primers, as indicated. Primer 2 is reverse complemented to primer 3, and primer 4 is reverse complemented to primer 5.

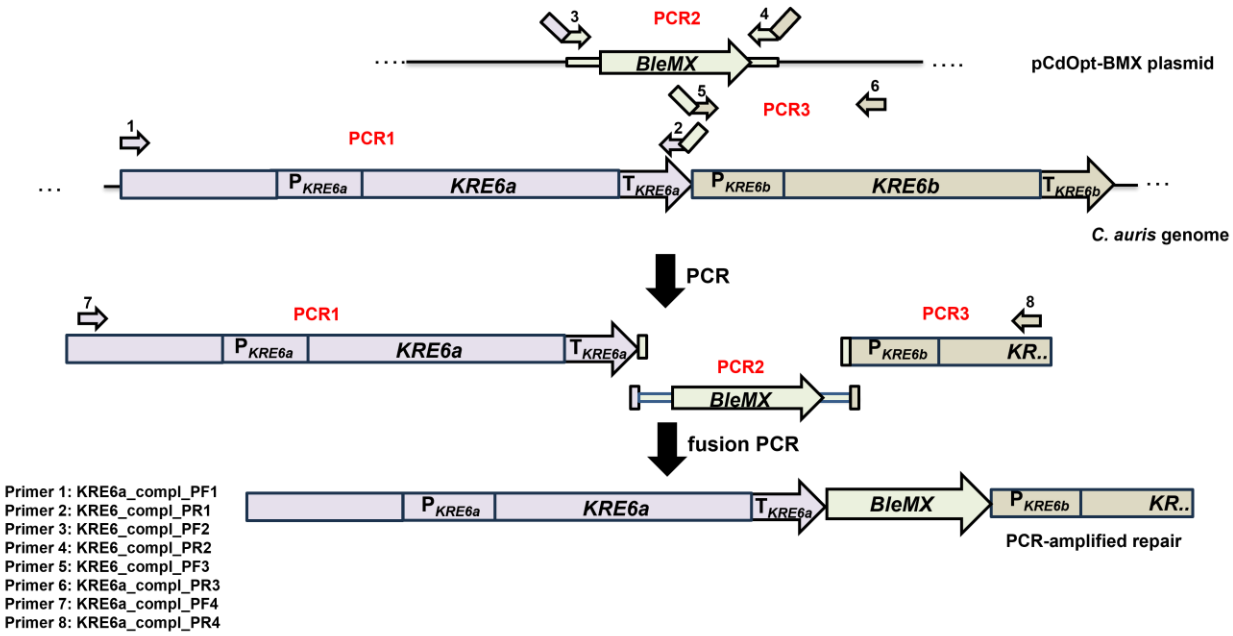

Supplementary Figure 10. Construction of the *C. auris kre6a*Δ::*KRE6a* strain. Schematic view of designed fusion PCRs to obtain a PCR-amplified repair fragment to complement *KRE6a*. The fragment contains *BleMX*, *KRE6a* promotor (P_{KRE6a}), *KRE6a* ORF, *KRE6a* terminator (T_{KRE6a}) and downstream region of *KRE6a*. The fusion PCRs were carried out with overlapping primers as indicated. Primer 2 is reverse complemented to primer 3, and primer 4 is reverse complemented to primer 5.

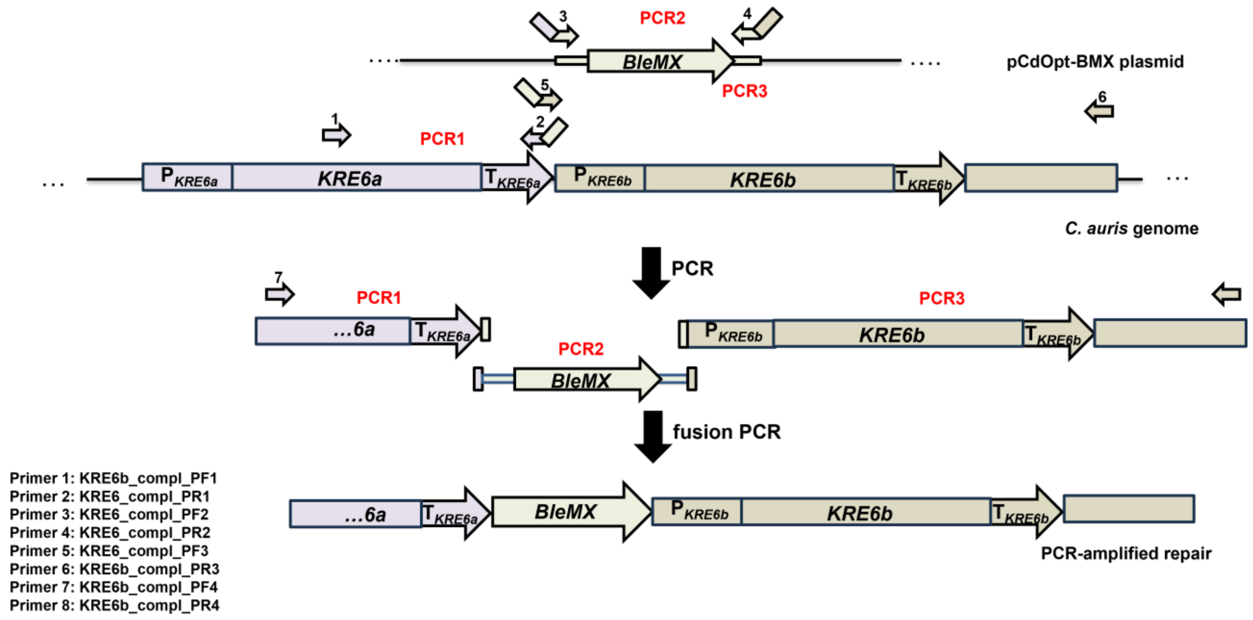

Supplementary Figure 11. Construction of the *C. auris kre6b*Δ::*KRE6b* strain. Schematic view of designed fusion PCRs to obtain a PCR-amplified repair fragment to complement *KRE6b*. The fragment contains *BleMX*, *KRE6b* promotor (P_{KRE6b}), *KRE6b* ORF, *KRE6b* terminator (T_{KRE6b}) and downstream region of *KRE6b*. The fusion PCRs were carried out with overlapping primers as indicated. Primer 2 is reverse complemented to primer 3, and primer 4 is reverse complemented to primer 5.

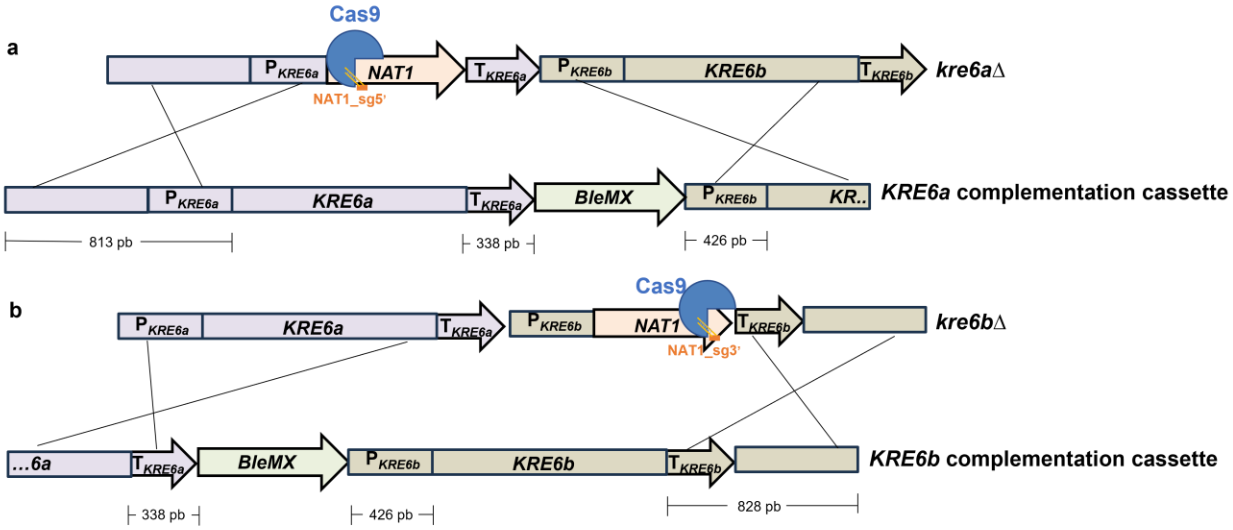

Supplementary Figure 12. Principles of construction of complement strains. (**a**) *kre6a*Δ::*KRE6a* and (**b**) *kre6b*Δ::*KRE6b.* Each complementation cassette was designed to target the endogenous locus of the gene of interest (*KRE6a* or *KRE6b*), replacing the selective marker *NAT1* (nourseothricin resistance) with *BleMX* (zeocin resistance). Guide RNAs were used to specifically target the *NAT1* cassette.

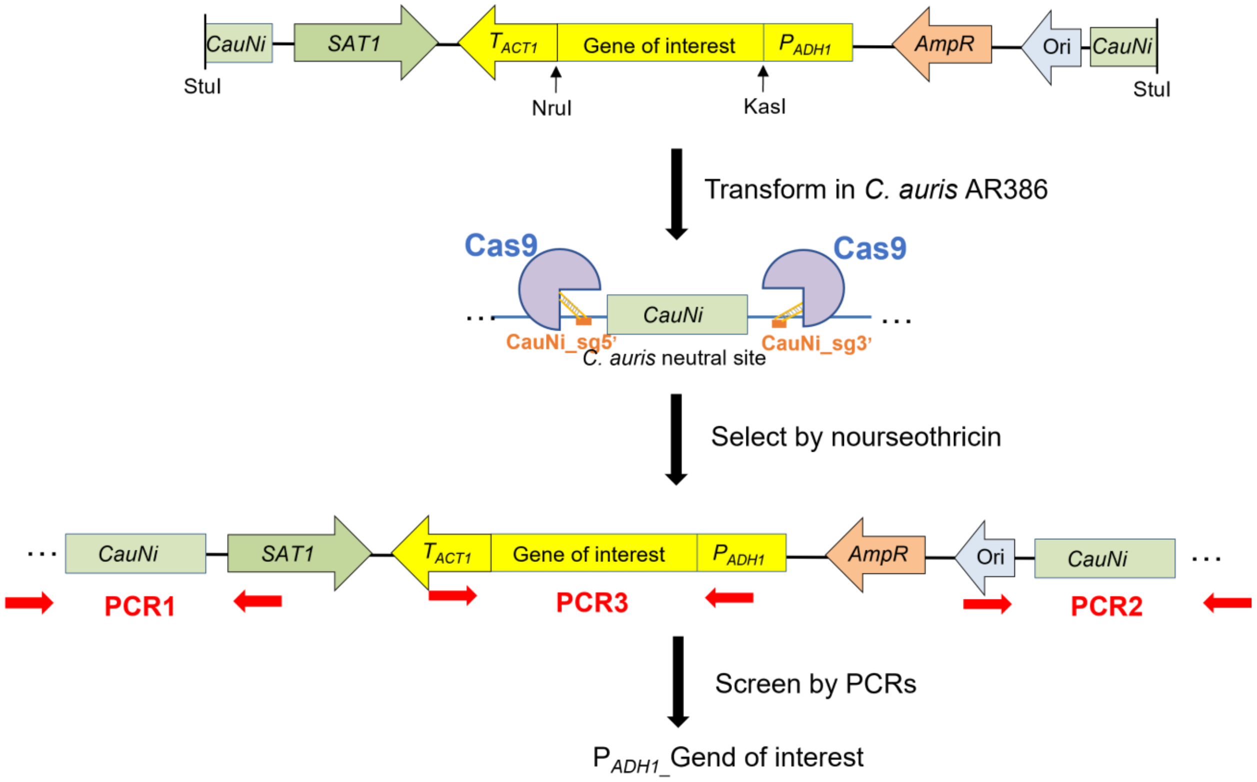

Supplementary Figure 13. Construction of the *C. auris* P$_{ADH1}$_*KRE6a* and P$_{ADH1}$_*KRE6b* strains. Schematic view of the overexpression system in *C. auris*. The plasmid contains the promotor P$_{ADH1}$, the gene of interest (*KRE6a* or *KRE6b*), the terminator of T$_{ACT1}$, the *SAT1* cassette (nourseothricin resistance) and the *C. auris* neutral site *CauNi*. The restriction sites KasI and NruI were used to insert the nucleotide sequences of gene of interest. The plasmid was linearized by StuI and transformed via electroporation in the wild-type AR386 strain with CRISPR-Cas9 method, by targeting the upstream and the downstream regions of *CauNi* with the nucleotide-specific guide RNAs CauNi_sg5' and CauNi_sg3'. The transformants were screened by performing three PCRs to verify the proper integration of the plasmid in the genome of *C. auris*. Source data are provided as a Source Data file.

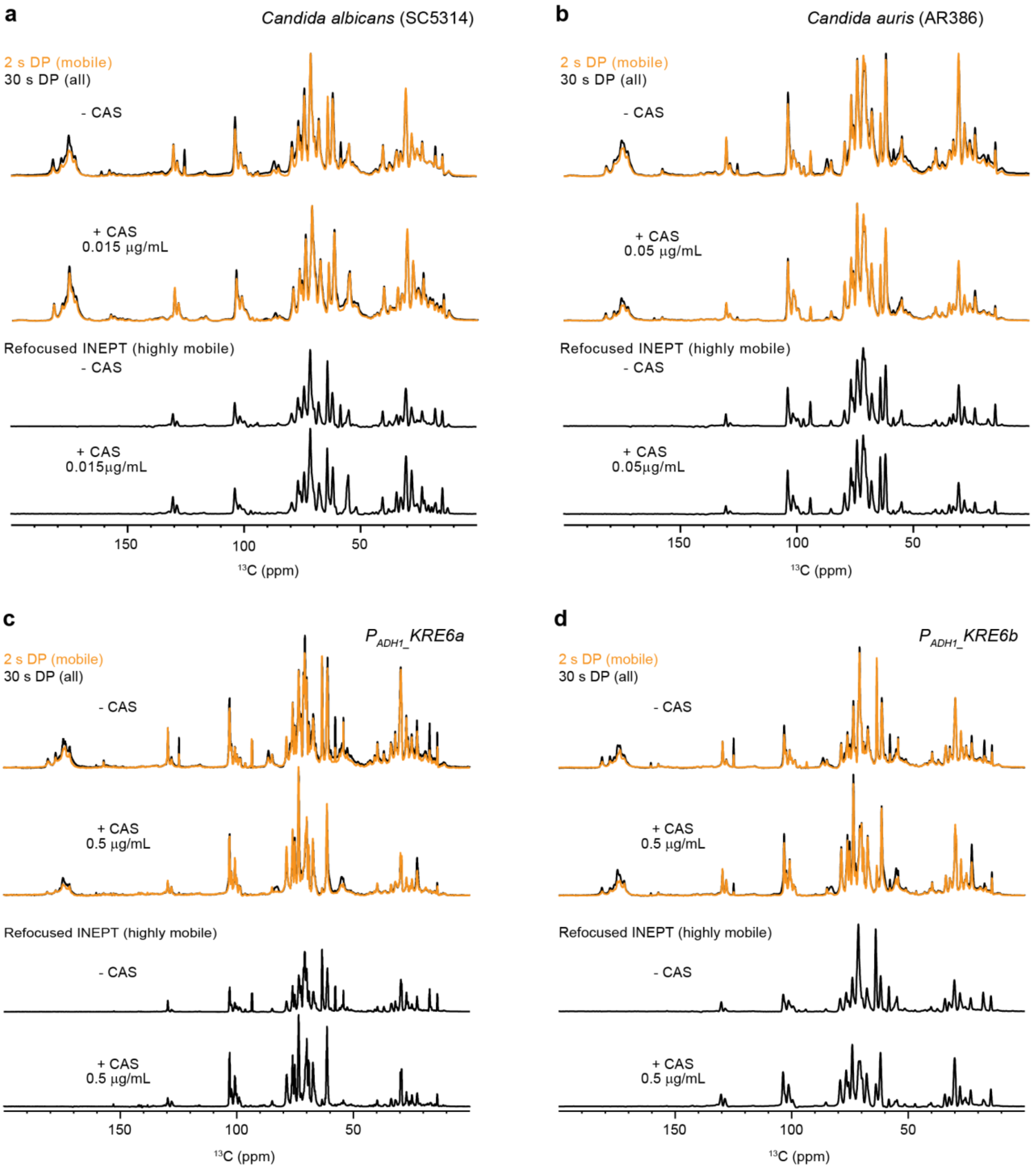

Supplementary Figure 14. Protein and lipid content in the mobile phase of *C. auris* cell walls. 1D ^{13}C spectra of (**a**) *C. albicans* and (**b**) *C. auris* with and without CAS treatment. The spectra include 1D ^{13}C 2 s DP for detecting mobile molecules, 30 s DP for quantitatively detecting all molecules, and 1D ^{13}C refocused INEPT for detecting highly mobile molecules in *C. albicans* and *C. auris*. The same set of 1D ^{13}C spectra were also measured on (**c**) P_{ADH1}_*KRE6a* and (**d**) P_{ADH1}_*KRE6b* with and without CAS treatment. The spectra include 1D ^{13}C 2 s DP for detecting mobile molecules, 30 s DP for quantitatively detecting all molecules, and 1D ^{13}C refocused INEPT for detecting highly mobile molecules in *C. auris KRE6* mutants.

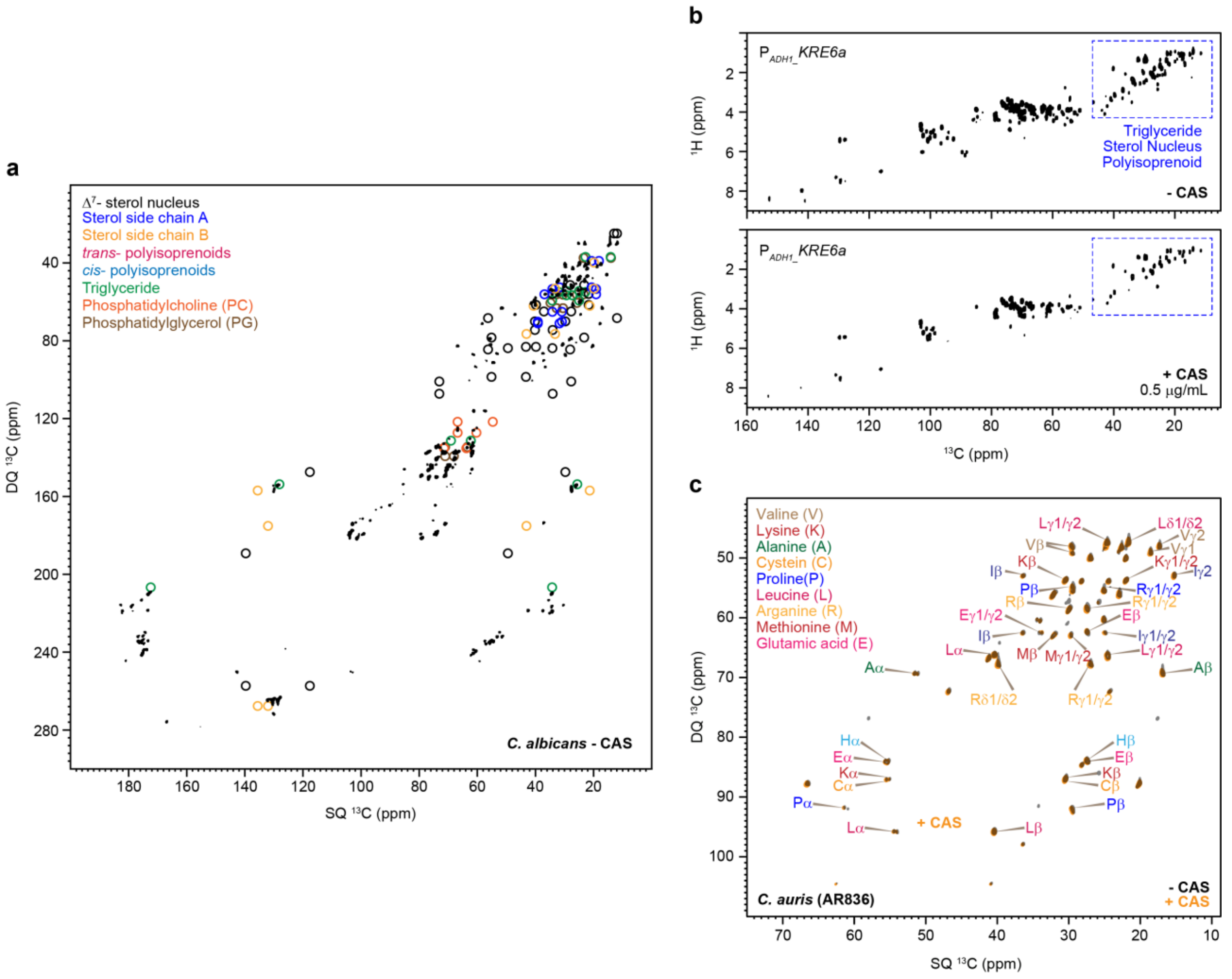

Supplementary Figure 15. Protein and lipids in the mobile fraction of *Candida* cell walls. (**a**) Overlay of 2D ^{13}C-^{13}C refocused DP J-INADEQUATE spectra of *C. albicans* with drug sample (black) with simulated spectra. The simulated spectra were plotted using the chemical shifts reported in recent NMR studies of lipid components (sterol, polyisoprenoid, and triglycerides) and model phospholipids POPC and POPG.[1-5] (**b**) 2D ^{1}H-^{13}C refocused INEPT spectra of *C. auris* P_{ADH1}_*KRE6a* with and without drug show the highly mobile protein, lipids, and polysaccharides. Protein and lipids are shown in blue color dashed boxes. (**c**) 2D ^{1}H-^{15}N HETCOR spectra showing amide and amine signals. (**c**) 2D ^{13}C-^{13}C refocused DP J-INADEQUATE spectra of *C. auris* AR386 showing signals of mobile proteins, with highly overlapping signals that indicate similar protein structures[6].

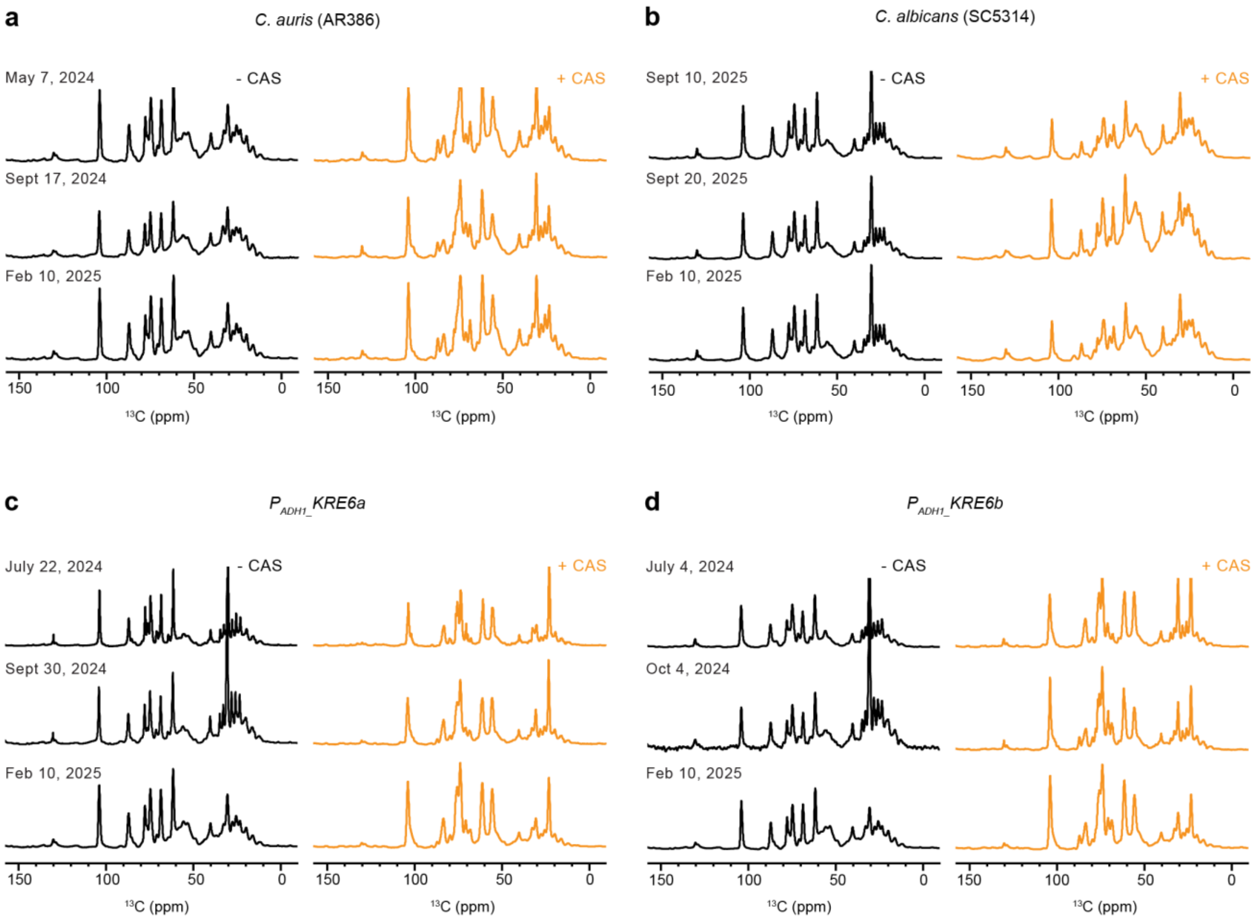

Supplementary Figure 16. 1D ^{13}C spectra collected at different time points. The carbohydrate spectral regions showed high reproducibility in 1D ^{13}C spectra collected at different time points for (**a**) C. auris AR 386, (**b**) C. albicans SC5314, (**c**) P_{ADH1}_*KRE6a* and (**d**) P_{ADH1}_*KRE6b*. For each sample, the spectra of both apo (black) and caspofungin-treated (orange) are shown. All Samples were measured on an 800MHz NMR spectrometer at 13.5KHz at 298K.

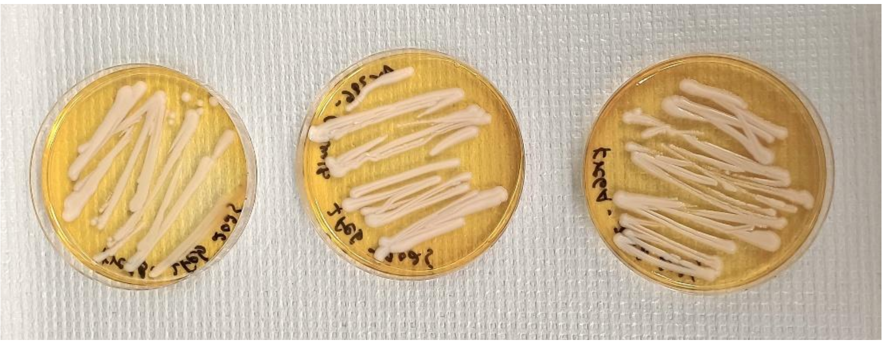

Supplementary Figure 17. Solid cultures showing cell viability after solid-state NMR experiments. From left to right are SC5314, AR386, and *KRE6aΔ*. *Candida* samples are regrown using fungal samples from the MAS rotor post-experiment. It confirms that at least a large portion of the *Candida* cells are viable and able to proliferate normally after the experiment[7].

Supplementary Table 1. ^{13}C and ^{15}N chemical shifts of polysaccharides in cell walls at room temperature and at DNP conditions. Branched (Br). Not applicable (/). Unidentified (-). Minor (m). The referencing scale is TMS scale.

Carbohydrate		C1	C2	C3	C4	C5	C6	CO	CH_3	N	Experimental method	Sample	Reference
Room-temperature solid-state NMR													
β-1,3-glucan		103.7	74.5	86.8	68.3	77.5	61.2	/	/	/	53 ms CORD	*C. albicans* apo	Shim *et al*. 2007 [8]
	m	103.6	74.6	85.2	68.3	77.4	61.1	/	/	/		*C. albicans*, *C. auris*	Fairweather *et al*. 2009 [9]
	m	103.3	74.4	85.3	68.3	77.5	61.1	/	/	/			Saito *et al*. 1979 [10]
Chitin		103.8	55.0	73.2	83.2	75.8	60.9	174.9	22.9	122.3	53 ms CORD	*C. albicans*	Fernando *et al*. 2021 [11]
		101.5	55.2	72.4	84.5	75.1	60.2	175.7	23.2	122.3		*C. auris*	
β-1,3,6-glucan	Br	103.1	73.2	85.5	68.7	75.7	69.2	/	/	/	Refocused DP J-INADEQUTE	*C. albicans*, *C. auris*	Lowman *et al*. 2011 [12]
β-1,6-glucan		103.9	74.2	76.3	70.6	75.4	69.6	/	/	/			
α-1,2-Mannan	a	101.4	79.1	71.0	67.5	73.8	61.9	/	/	/		all samples	Latgé *et al*.1994 [13] Chakraborty *et al*. 2021 [14]
	b	99.2	79.1	71.4	67.8	74.1	61.9	/	/	/			
α-1,6-Mannan		102.7	70.8	73.8	67.7	71.2	67.3	/	/	/			
Galactose	a	90.2	74.2	-	-	-	-	/	/	/		*C. albicans* apo, CAS	Fontaine *et al*. 2011 [15]
	b	96.8	74.9	76.8	70.6	72.3	61.5	/	/	/		all samples	
	d	94.8	72.0	-	-	-	-	/	/	/			
Glucose	a	92.9	72.1	73.8	70.5	72.0	63.9	/	/	/		all samples	
	b	96.8	74.9										
MAS-DNP													
β-1,3-glucan		103.0	74.1	85.0<br>83.9	68.1	77.8	61.6				DNP 5 ms PAR	*C. auris,* apo, CAS	
Chitin		102.5	55.2	72.8	81.9	74.8	59.1	175.9<br>174.9<br>172.9	22.3				
α-1,2-Mannan		101.4	81.1	70.0	66.3	72.3	62.5						
α-1,2-Mannan (m)		101.9	81.9										

Supplementary Table 2. Chemical shifts of *C. albicans* and *C. auris* polysaccharides from ^{1}H-detection. ^{13}C and ^{1}H chemical shifts are shown in top and bottom rows, respectively. TMS scale for ^{13}C. DSS for ^{1}H. Sites with ambiguity: underlined.

Carbohydrates		forms	C1	C2	C3	C4	C5	C6	Reference
Rigid molecules	β -1,3-glucan (B)		103.9<br>5.0	74.44<br>3.74	86.89<br>3.71	68.52<br>3.71	77.6<br>3.54	61.54<br>3.94	Chakraborty *et al.* 2021 [14]
	Chitin (Ch)		---	55.5<br>4.4	---	83.3<br>3.8	75.8<br>3.6	---	Fernando *et al.* 2021 [11]
Mobile molecules	β-1,3-glucan (B)	a	103.6<br>4.55	74.1<br>3.3	85.2<br>3.75	69.3<br>4.2	76.0<br>3.52	61.6<br>3.75	Shim *et al.* 2007 [8]<br>Fairweather *et al.* 2009 [9]<br>Saito *et al.* 1979 [10]
		b	---	74.65<br>3.4	85.12<br>4.12	69.9<br>4.2	---	61.95<br>3.9	
		c	---	74.49<br>3.52	86.74<br>4.28	71.59<br>4.43	---	62.5<br>3.8	
	β-1,3,6-glucan (Br)		---	---	84.7<br>4.3	---	75.44<br>3.6	69.6<br>3.8	Lowman *et al.* 2011 [12]
	β-1,6-glucan (H)		103.54<br>4.52	74.15<br>3.3	76.3<br>3.5	70.4<br>3.89	---	69.9<br>3.87	
	α-1,6-Mannan ($Mn^{1,6}$)		102.7<br>5.14	70.8<br>3.4/4.0	73.8<br>3.34/3.7	67.7<br>3.6	70.8<br>3.8	---	Latge *et al.* 1994 [13]<br>Chakraborty *et al.* 2021 [14]
	α-1,2-Mannan ($Mn^{1,2}$)	a	101.4<br>5.28	79.19<br>4.11	70.89<br>3.94	67.8<br>3.7	73.98<br>3.74	62.1<br>3.9	Kuraoka *et al.* 2021 [16]
		b	98.97<br>5.08	79.51<br>4.0	71.19<br>3.95	67<br>3.71	73.4<br>3.68	61.98<br>3.94	
		c	100.74<br>5.16	78.22<br>4.28	70.12<br>3.94/4.19	68.09<br>3.6	73.98<br>3.75	61.73<br>3.77	
		d	101.45<br>5.36	79.12<br>4.11	70.66<br>3.92	67.88<br>3.6	73.61<br>3.64	62.0<br>3.67	Kuraoka *et al.* 2018 [17]
		e	102.7<br>5.04	79.13<br>3.91/4.1	71.2<br>3.8	67.7<br>3.6	74.23<br>3.34	61.9<br>3.7, 3.9	Kuraoka *et al.* 2021[16]
	Galactose/Glucose or their derivatives (Gl)	a	90.04<br>5.9	85.4<br>4.1/3.7	74.5<br>4.34	70.27<br>4.2	---	61.84<br>3.8, 3.9	Fontaine *et al.* 2011 [15]
		b	97.06<br>5.45	71.08<br>4.02	74.36<br>3.2	67.03<br>3.72	---	61.82<br>3.7, 3.9	Archbald et al. 1981[18]
		c	94.66<br>4.9	72.5<br>3.8	73.59<br>3.65	67.42<br>3.58	---	61.9<br>3.7, 3.8	Archbald et al. 1981 [18]<br>Fontaine *et al.* 2011 [15]
		d	94.98<br>5.18	72.5<br>3.81	71.3<br>3.85	67.6<br>3.66	---	61.9<br>3.7.3.8	Archbald et al. 1981 [18]<br>Fontaine *et al.* 2011[15]
		e	89.24<br>6.05	86.74<br>4.2	74.8<br>4.34/3.4	71.39<br>4.4	---	62.59<br>3.8, 3.9	Fontaine *et al.* 2011 [15]
	Glucose (Glc)	a (α)	93.07<br>5.237	72.4<br>3.5	72.4<br>3.5	70.4<br>3.4	---	61.56<br>3.84, 3.76	Roslund et al. 2008<br>Archbald *et al.* 1981 [18]
		b (β)	96.84<br>4.65	74.9<br>3.25	76.58<br>3.47	70.48<br>3.4	---	61.79<br>3.9,3.73	

Supplementary Table 3. The average cell wall thickness of comparable-sized yeast cells. Results are described as the average and standard deviation of 100 measurements for each sample. A source data file is provided to document each single reading.

Sample	Strain	Average cell wall thickness (nm)	
		apo	+CAS
C. albicans	SC5314	140±11	185±16
C. auris	AR836	158±11	162±13
	I.3	70±17	75±17
	P_{ADH1}_*KRE6a*	114±23	183±28
	P_{ADH1}_*KRE6b*	116±26	175±21

Supplementary Table 4. The molar composition of rigid polysaccharides. The numbers are estimated using integrals (volume) of cross peaks in 2D ^{13}C-^{13}C 53 ms CORD spectra. The average integrals of cross-peaks of each polysaccharide are shown. Error bars are standard errors.

Sample		Polysaccharide					
Strain	Condition	β-1,3-glucan			Chitin	β-1,6-glucan	Mannan
		a	b	c			
C. albicans (SC5314)	apo	87±11	1.1±0.2	6±1	5±2	1.1±0.1	ND
	+CAS	46±6	0.4±0.2	ND	44±8	2.6±0.2	7±2
C. auris (AR386)	apo	63±6	6±2	11±3	18±4	1.3±0.1	1.1±0.2
	+CAS	37±5	1.0±0.3	3.0±0.8	48±7	5.0±0.4	5.5±0.8
C. auris (I.3)	apo	77±9	ND	3±2	5±2	9.1±0.7	6±2
	+CAS	66±9	ND	5±2	12±2	5.1±0.3	12±2
C. auris (P_{ADH1}_*KRE6a*)	apo	85±11	ND	2±1	8±2	5.2±0.8	ND
	+CAS	ND	ND	ND	80±13	7.1±0.9	13±2
C. auris (P_{ADH1}_*KRE6b*)	apo	84±11	ND	ND	12±2	4.1±0.5	ND
	+CAS	ND	ND	ND	82±16	10±1	8±1
C. auris (*kre6a*Δ)	apo	95±13	ND	ND	ND	5±2	ND
	+CAS	12±2	ND	ND	79±13	4±1	5.0±0.8
C. auris (*kre6b*Δ)	apo	99.3±9.7	ND	ND	ND	0.7±0.3	ND
	+CAS	38±6	ND	ND	49±10	7±2	6±1

The area of the following well-resolved cross peaks 53 ms CORD spectra are used:
β-1,3 (a): the average of C1-C2/3/4/5 and C3-C2/4/5/6.
β-1,3 (b): the average of C1-C5, C2-5, C4-5, and C6-5.
β-1,3 (c): the average of C3-C2/4/5/6.
β-1,6: the average of C3/5-C4 and C5-C6.
Chitin: the average of C1-2/4/5, C3-C2, C4-C2/3/5, C5-C2, and C6-C2.
Mannan: the average of C2-C5 and C2-C3.

Supplementary Table 5. The molar composition of mobile polysaccharides. The numbers are estimated using integrals (volume) of cross peaks in 2D ^{13}C-^{13}C refocused DP-J INADEQUATE spectra. The average integrals of cross-peaks of each polysaccharide are shown. Error bars are standard errors of the peak integrals.

Sample		Polysaccharide									
		β-glucan			Mannan^{1,2}		Mannan^{1,6}	Galactose			
Strain	Condition	β-1,6-glucan	β-1,3-glucan	β-1,3,6-glc	a	b		a	b	c	d
C. albicans (SC5314)	apo	30±6	33±7	1.2±0.3	14±2	5±1	8.9±0.8	4.0±0.3	2.0±0.3	1.1±0.5	1.1±0.6
	+CAS	19±3	15±4	2.6±0.3	24±3	5±1	21±2	7.8±0.5	1.7±0.2	0.9±0.2	2.3±0.5
C. auris (AR386)	apo	33±4	24±2	1.5±0.3	9±1	2.9±0.2	6±2	4.1±0.3	7±1	4±1	8±2
	+CAS	35±3	20±1	0.7±0.2	14±1	2.8±0.1	9.1±0.9	2.2±0.2	7±1	4±1	6±2
C. auris (I.3)	apo	33±4	31±5	2.5±0.2	11±2	6.3±0.6	5±2	1.2±0.2	4.0±0.5	5.3±0.5	1.0±0.7
	+CAS	30±2	22±4	3.1±0.3	15±2	6±1	6±2	0.65±0.04	2.9±0.6	0.7±0.7	13±1
C. auris (P$_{ADH1}$ *KRE6a*)	apo	32±2	26±5	4.6±0.3	12±1	4.4±0.7	7±1	1.24±0.06	7.3±0.9	4.7±0.7	1.4±0.3
	+CAS	36±7	19±6	2.8±0.3	24±3	5.5±0.5	11±3	0.9±0.1	0.36±0.07	ND	0.7±0.2
C. auris (P$_{ADH1}$ *KRE6b*)	apo	32±4	16±4	6.3±0.4	21±2	8.7±0.8	13.8±0.9	1.32±0.07	0.51±0.03	0.45±0.08	0.9±0.1
	+CAS	35±6	21±5	2.5±0.2	23±3	5.4±0.7	11±1	1.2±0.2	ND	0.12±0.03	ND
C. auris (*kre6a*Δ)	apo	19±3	44±7	5.0±0.8	7±1	4.0±0.5	8±1	ND	9.6±3.2	ND	3.4±0.4
	+CAS	34±7	36±12	4±0.6	11±3	5.0±0.9	10±3	ND	ND	ND	ND
C. auris (*kre6b*Δ)	apo	23±5	40±14	7±1	5±1	3.0±0.8	6±2	ND	11±3	ND	5±1
	+CAS	53±16	24±8	4.0±0.9	7.0±1.2	4±1	8±2	ND	ND	ND	ND

The area of the following well-resolved cross peaks refocused DP-J INADEQUATE spectra are used:
β-1,6: the average of C3, C4, C5, and C6.
β-1,3; the average of C1, C2, C5, and C6
β-1,3, 6: the average of C2, C3, and C4.
Mannan^{1,2} (a and b): the average of C1 and C2.
Mannan^{1,6}: the average of C1 and C2.
Galactose (a, b, c, and d): the average of C1 and C2.

Supplementary Table 6. Water-edited intensities of polysaccharide carbon sites. The intensity ratios are obtained by comparing the peak intensities in water-edited and control 2D spectra. The average values for each molecule in each sample are highlighted in bold. Error bars are standard deviations propagated from NMR signal-to-noise ratios.

Polysaccharide	Cross-peak	*C. albicans* (SC5314)		*C. auris* (AR386)	
		apo	+CAS	apo	+CAS
β-1,3-glucan	B1-3	0.8±0.1	-	0.6±0.1	0.28±0.06
	B1-5	0.7±0.1	0.8±0.3	0.5±0.1	0.25±0.05
	B1-2	0.73±0.08	0.6±0.1	0.54±0.07	0.19±0.02
	B1-4	0.7±0.1	0.7±0.2	0.6±0.1	0.29±0.05
	B1-6	0.7±0.1	0.5±0.1	0.7±0.1	0.17±0.04
	B3-1	0.7±0.1	-	0.7±0.2	0.26±0.04
	B3-5	0.7±0.2	0.8±0.3	0.6±0.2	0.26±0.06
	B3-2	0.8±0.1	0.6±0.2	0.5±0.1	0.22±0.05
	B3-4	0.7±0.1	0.9±0.3	0.5±0.1	0.24±0.06
	B3-6	0.8±0.2	0.8±0.4	0.5±0.2	0.29±0.07
	B5-1	0.8±0.1	0.8±0.2	0.6±0.1	0.23±0.04
	B5-3	0.8±0.2	0.8±0.3	0.6±0.2	0.22±0.05
	B5-2	0.7±0.1	0.5±0.1	0.6±0.1	0.15±0.04
	B5-4	0.7±0.1	0.8±0.2	0.6±0.1	0.25±0.03
	B5-6	0.67±0.07	0.7±0.1	0.56±0.08	0.21±0.03
	B2-1	0.66±0.07	0.61±0.09	0.58±0.06	0.20±0.02
	B2-3	0.8±0.2	-	0.7±0.1	0.23±0.05
	B2-5	0.9±0.2	0.7±0.2	0.6±0.1	0.19±0.04
	B2-4	0.7±0.1	0.7±0.2	0.60±0.09	0.23±0.04
	B2-6	0.6±0.1	0.48±0.09	0.5±0.1	0.16±0.03
	B4-1	0.64±0.09	0.7±0.2	0.51±0.09	0.24±0.04
	B4-3	0.5±0.1	0.7±0.2	0.6±0.1	0.25±0.05
	B4-5	0.7±0.1	0.8±0.2	0.6±0.1	0.27±0.04
	B4-2	0.61±0.08	0.6±0.1	0.50±0.09	0.19±0.03
	B4-6	0.71±0.08	0.6±0.1	0.50±0.09	0.26±0.04
	Average	0.71	0.68	0.57	0.23
Chitin	Ch1-4	0.5±0.2	0.3±0.1	0.6±0.2	0.06±0.04
	Ch1-5	-	0.6±0.1	0.36±0.06	0.17±0.02
	Ch1-2	-	0.17±0.07	0.3±0.2	0.08±0.03
	Ch4-1	-	0.2±0.1	-	0.07±0.04
	Ch4-5	0.7±0.4	0.3±0.2	-	0.08±0.02
	Ch4-2	-	-	-	0.08±0.04
	Ch5-4	0.3±0.1	0.28±0.07	0.3±0.1	0.04±0.02
	Ch5-2	-	0.22±0.09	-	0.05±0.03
	Ch3-4	0.6±0.3	0.25±0.09	0.2±0.1	0.08±0.03
	Ch3-2	-	0.3±0.1	0.2±0.1	0.07±0.02
	Ch2-1	-	0.16±0.06	0.1±0.1	0.10±0.04
	Ch2-4	-	0.19±0.09	0.3±0.4	0.06±0.04
	Ch2-5	-	0.25±0.09	0.1±0.2	0.04±0.03
	Ch2-3	-	0.25±0.05	0.2±0.2	0.09±0.03
	Ch2-1	-	0.2±0.1	-	0.09±0.07
	Average	0.53	0.27	0.27	0.08

Supplementary Table 7. ^{1}H-$T_{1\rho}$ and ^{13}C-T_1 relaxation times of polysaccharides in cell walls. Data are shown for the *C. albicans* and *C. auris* samples, with and without treatment by caspofungin. The average values for each molecule in each sample are highlighted in bold. The data were measured using 1D ^{13}C relaxation experiments. The data are fit using single exponential equations: $I(t) = e^{-t/T_1}$. Error bars are standard deviations of the fit parameters.

Polysaccharide	Chemical shift	*C. albicans* (SC5314)				*C. auris* (AR386)			
		apo		+CAS		apo		+CAS	
		^{1}H-$T_{1\rho}$ (ms)	^{13}C-T_1 (s)	^{1}H-$T_{1\rho}$ (ms)	^{13}C-T_1 (s)	^{1}H-$T_{1\rho}$ (ms)	^{13}C-T_1 (s)	^{1}H-$T_{1\rho}$ (ms)	^{13}C-T_1 (s)
β-1,3-glucan	86.4	14.5±0.6	1.47±0.03	6.6±0.6	1.19±0.06	9.4±0.6	1.36±0.03	8.1±0.5	1.25±0.02
	77.1	14.5±0.9	1.54±0.04	7.0±0.5	1.03±0.07	9.1±0.5	1.40±0.03	7.0±0.6	1.25±0.02
	74.4	14.8±0.7	1.58±0.02	13.1±0.9	1.14±0.09	9.5±0.5	1.63±0.05	13±1	1.57±0.07
	68.7	18.8±0.5	1.56±0.03	7.5±0.6	0.92±0.05	9.4±0.4	1.47±0.03	9.4±0.6	1.20±0.04
	61.3	13.7±0.9	-	9.0±0.7	-	9.8±0.5	-	10.2±0.8	-
	Average	15.3	1.5	8.6	1.1	9.4	1.5	9.5	1.3
Chitin	83	-	-	17.0±0.9	4.1±0.2	16.5±0.9	2.4±0.3	17.7±0.9	3.0±0.2
	75.7	-	-	16±1	2.1±0.2	9.9±0.9	2.1±0.3	13.5±0.8	2.4±0.2
	72.9	8.5±0.8	0.5±0.1	14±1	-	-	-	-	1.8±0.2
	55.5	6.5±0.6	0.8±0.1	11.0±0.7	2.6±0.2	12.5±0.4	2.5±0.2	15.6±0.8	2.5±0.2
	Average	7.5	0.65	14.5	2.9	13.0	2.3	15.6	2.4

Supplementary Table 8. Primers and RNAs guides used in this study.

Primer / guide RNA	Aim	Sequence (5' → 3')
	***KRE6a/b* deletion**	
KRE6_del_PF1	*KRE6a/b* and *KRE6a* deletion cassette construction, *KRE6a* deletion verification	TAT GCA GTA CGC GTG AAA ACT GC
KRE6_del_PR1	*KRE6a/b* and *KRE6a* deletion cassette construction	GTA TTC TGG GCC TCC ATG TCA GCG TTT GGG GAT GAA GAT GG
KRE6_del_PF2	*KRE6a/b* and *KRE6a* deletion cassette construction	CCA TCT TCA TCC CCA AAC GCT GAC ATG GAG GCC CAG AAT AC
KRE6_del_PR2	*KRE6a/b* and *KRE6b* deletion cassette construction	GCA AAA CCA AGC GAA GGA ATA GCC AGT ATA GCG ACC AGC ATT CAC
KRE6_del_PF3	*KRE6a/b* and *KRE6b* deletion cassette construction	GTG AAT GCT GGT CGC TAT ACT GGC TAT TCC TTC GCT TGG TTT TGC
KRE6_del_PR3	*KRE6a/b* and *KRE6b* deletion cassette construction	TTA CAT GGC CAT GAA AAT GGC GC
KRE6_del_PF4	*KRE6a/b* and *KRE6a* deletion cassette construction	AAA ATC TGA GGC TGT GTG TCG C
KRE6_del_PR4	*KRE6a/b* and *KRE6b* deletion cassette construction	AAG TCG ATC CGA GTC AGG TG
KRE6a_del_PR2	*KRE6a* deletion cassette construction	CCA CAA CGT CAA GTT GGG GTC AGT ATA GCG ACC AGC ATT CAC
KRE6a_del_PF3	*KRE6a* deletion cassette construction	GTG AAT GCT GGT CGC TAT ACT GAC CCC AAC TTG ACG TTG TGG
KRE6a_del_PR3	*KRE6a* deletion cassette construction	TCC CGA TCC ATG CTA CCT TG
KRE6a_del_PR4	*KRE6a* deletion cassette construction	CCT TCT ACT TCT CTG CCT CTC
KRE6b_del_PF1	*KRE6b* deletion cassette construction	CCA TCA CCA TCA CCT TCA ATG C
KRE6b_del_PR1	*KRE6b* deletion cassette construction	GTA TTC TGG GCC TCC ATG TCA GCG AGC AGC TAT GAG GAA AAA G
KRE6b_del_PF2	*KRE6b* deletion cassette construction	CTT TTT CCT CAT AGC TGC TCG CTG ACA TGG AGG CCC AGA ATA C
KRE6b_del_PF4	*KRE6b* deletion cassette construction	TTG TTT TTG TGG CGC CAG CC
KRE6_del_verif_PF	*KRE6a/b* deletion verification	AGG CTT AGT GAG AAA CCC CTA C
NatR_verif_PR	*KRE6a/b* deletion and *KRE6b* deletion verification	AGC ATC ACC TGG AAC AGA AGT TC
KRE6_PF	*KRE6a/b* deletion verification	AGA TAG CGC CCA TGG ACA TC
KRE6_PR	*KRE6a/b* deletion and *KRE6a* deletion verification	TAG TTT CAG ACG ACC CAC CTC
NAT1_743_PF	*KRE6a* deletion verification	GTG CTG GTC ATT TGT GGT TG
KRE6a_PR	*KRE6a* deletion verification	ATG ATG CCT TGG CTT CGA CTG
KRE6a_PF4	*KRE6b* deletion verification	ATC CAG CAA GCT GTT TCG GG
KRE6b_PF2	*KRE6b* deletion verification	GAG GTG GGT CGT CTG AAA CTA
KRE6b_PR(sybrgreen)	*KRE6b* deletion verification	CTT GAA CCC TGC CAT CTC CC
KRE6_del_sg5'	*KRE6a/b* deletion and *KRE6a* guide RNA	ACU GUG UUC UGG GAC UGU GG
KRE6_del_sg3'	*KRE6a/b* deletion and *KRE6b* deletion guide RNA	AUG CCG AAG AAC AAA CUC AG
	***KRE6a/b* overexpression**	
KRE6a_PF_KasI	*KRE6a* overexpression plasmid construction	ACA CTG GCG CCA TGG TCC GTG ACT TGA CCT C
KRE6a_PR_NruI	*KRE6a* overexpression plasmid construction	ACA CTT CGC GAT CAA CAG TCG TAG GCC AAC TTG
KRE6b_PF_KasI	*KRE6b* overexpression plasmid construction	ACA CTG GCG CCA TGT CCC ACA GAG ACC TCA C
KRE6b_PR_NruI	*KRE6b* overexpression plasmid construction	ACA CTT CGC GAC TAG CAA CCA CTG AGT TTG TTC TT
pjli8_ADH1_PF	*KRE6a/*b overexpression plasmid sequencing	AGC AAC ACC GGT GGA ATT TCC
KRE6a_PF2	*KRE6a* overexpression plasmid sequencing	TTG CAC AAC CCA GAC CCA ATT G
KRE6a_PF3	*KRE6a* overexpression plasmid sequencing	GCC ACA ATT TGT TCT ACC GCT C
KRE6a_PF4	*KRE6a* overexpression plasmid sequencing	ATC CAG CAA GCT GTT TCG GG
KRE6b_PF2	*KRE6b* overexpression plasmid sequencing	GAG GTG GGT CGT CTG AAA CTA
KRE6b_PF3	*KRE6b* overexpression plasmid sequencing	GAA CTT TCT ACG ATG GCG ACG
KRE6b_PF4	*KRE6b* overexpression plasmid sequencing	GAC ACT CTC AAA ACT GGT GTG G
CauNi_sg5'	*KRE6a/b* overexpression guide RNA	CCC GGA GAU ACA CGG CGC CG
CauNi_sg3'	*KRE6a/b* overexpression guide RNA	GCU GCA AAA UAA GGC CAG AG
	**RT-PCR**	
ACT1_F(sybrgreen)	RT-PCR	GAA GGA GAT CAC TGC TTT AGC C
ACT1_R(sybrgreen)	RT-PCR	GAG CCA CCA ATC CAC ACA G
KRE6a_PF(sybrgreen)	*KRE6a* RT-PCR	TGA CGT GGT ATG TGG GAA GC
KRE6a_PR(sybrgreen)	*KRE6a* RT-PCR	GGG CTC TTT GGA AAT GCG TC
KRE6b_PF(sybrgreen)	*KRE6b* RT-PCR	TCC TGA AGA CTA CCC GAC GT
KRE6b_PR(sybrgreen)	*KRE6b* RT-PCR	CTT GAA CCC TGC CAT CTC CC
FKS1_PF(sybrgreen)	*FKS1* RT-PCR	GTA TGG GTT ACA TGG CCG CT
FKS1_PR(sybrgreen)	*FKS1* RT-PCR	GCA AGA ATG AAG TCA CCG GC
CHS1_PF(sybrgreen)	*CHS1* RT-PCR	CCA GGA GAA ACG GGC AGA AA
CHS1_PR(sybrgreen)	*CHS1* RT-PCR	TAA CCC GTA GAG CCA ATC GC

Supplementary Table 9. ^{13}C and ^{15}N Solid-state NMR experimental parameters for fungal cell wall characterization. T = sample temperature; B_0 = magnetic field; ν_{MAS} = MAS frequency; ns = number of scans; d_1 = recycle delay between scans; $t_{1, max}$ = maximum t_1 evolution time (for indirect dimension); $t_{1, inc}$ = increment for t_1 (for indirect dimension) evolution time; τ_{dw} = dwell time during direct FID acquisition; τ_{acq} = maximum acquisition time during direct FID detection; τ_{XY} = cross-polarization contact time during CP from channel X to channel Y; $\nu_{1H, dec}$ = dipolar decoupling field strength**.** DNP experiments are marked with asterisks. Spin diffusion (SD).

Experiment	NMR Parameters																Samples
	T (K)	B_0 (T)	ν_{MAS} (kHz)	ns	d1 (s)	$t_{1, max}$ (ms)	$t_{1, inc}$ (µs)	τ_{dw} (µs)	τ_{acq} (ms)	τ_{HC} (ms)	τ_{HN} (ms)	τ_{NC} (ms)	τ_{SD} (ms)	τ_{mix} (ms)	$\nu_{1H\ dec}$ (kHz)	Time (h)	
**Identification and quantification of polysaccharides**																	
1D ^{13}C CP	298	18.8	13.5	256	1.8			7	18	1					83	0.1	C. al apo C.al CAS (SC5314) C. au apo C.au CAS (AR386, P*ADH1*_*KR E6a*)
1D ^{15}N CP	298	18.8	13.5	128	2			10	16		1				83	0.1	
1D ^{13}C DP	298	18.8	13.5	32-64	2 or 30			7	29						100	0.1-0.5	
1D ^{13}C refocused INEPT	298	18.8	13.5	256	4			7	29					1.7 τ_J	71	0.3	
2D ^{13}C-^{13}C with CORD mixing	298	18.8	13.5	16	1.5	7	29	7.5	18	0.5				53 τ_{CORD}	83	3.4	
2D ^{13}C-^{13}C refocused DP J-INADEQUATE	298	18.8	13.5	8	1.5	10	20	7.5	19						83	3.4	
2D ^{15}N-^{13}C N(CA)CX with DARR mixing	298	18.8	13.5	64	1.6	7	180	7.5	18		0.6	5		100 τ_{DARR}	93	2.2	
2D ^{1}H-^{13}C refocused INEPT	298	18.8	13.5	4	2	11	50	7.5	23					1.7 τ_J	71	1.0	
2D ^{1}H-^{15}N HETCOR with SD	298	18.8	13.5	16	1.6	2	41	7.5	18		1		1		93	0.8	
**Estimation of site-specific hydration of polysaccharides**																	
2D ^{13}C-^{13}C water-edited	290	9.4	10	128-256	1.6	5	71	10	14	1			0, 4	50 τ_{PDSD}	71	16.4	
**Dynamics of polysaccharides**																	
1D ^{13}C-T_1	298	9.4	10	128-256	2			10	16	1					71	3.9	
1D ^{1}H-$T_{1\rho}$	298	9.4	10	256	2			10	14	1					71	2.0	
**Intermolecular interactions of polysaccharides**																	
* 2D ^{13}C-^{13}C with PAR mixing	92	14.1	8	4	5	7	33	7.5	15	0.5				5-20 τ_{PAR}	93	2.2	C. au apo C.au CAS

Supplementary Table 10. Parameters used for proton detection experiments. The CP based 2D hCH proton detection experiments were performed on 600 MHz (14.1 T) spectrometer with the MAS frequency of 60 kHz and 2D and 3D hCCH TOCSY (DIPSI-3) was performed on 800 MHz (18.8 T) with the MAS frequency of 13.5 kHz.

**Expt.**	**Samples**	**Temperature (K)**	**CP (µs)**		**D1**	**NS**	**td2**	**td1**	**td3**	**aq2 (ms)**	**aq1 (ms)**	**aq3 (ms)**	**Decoupling**	**Water suppression**	***J*-evolution (ms)**	**DIPSI-3 (ms)**	**Time (h)**
			t_{cp1}	t_{cp2}													
2D hCH	*C. albicans*<br>*C.auris*	304<br>300	900	100	2	64	1764	320	-	14.9	5.3	-	slpTPPM<br>(rf 20.280 kHz)	MISSISSIPI<br>(total duration)<br>100 ms<br>(rf 30 kHz)	-	-	11.4
2D & 3D hCCH TOCSY (DIPSI-3)	*C. albicans*<br>*C.auris*	296<br>295	-	-	1.89	8	2614	128	128	39.9	2.56	2.56	SPINAL-64<br>(rf 71.429 kHz)<br>WALTZ-16<br>(rf 10 kHz)	MISSISSIPI<br>(total duration)<br>40 ms<br>(rf 25.994 kHz	1.78 (τ_1)<br>1.19 (τ_2	25.5	2D: 0.6<br>3D: 68.8

Supplementary References

1. Chrissian, C. et al. Solid-state NMR spectroscopy identifies three classes of lipids in Cryptococcus neoformans melanized cell walls and whole fungal cells. *J. Biol. Chem.* **295**, 15083-15096 (2020).
2. Lamona, G. et al. Solid-state NMR molecular snapshots of Aspergillus fumigatus cell wall architecture during a conidial morphotype transition. *Proc. Natl. Acad. Sci. USA* **120** (2023).
3. Fernando, L.D. et al. Structural adaptation of fungal cell wall in hypersaline environment. *Nat. Commun.* **14**, 7082 (2023).
4. Suttiarporn, P. et al. Structures of Phytosterols and Triterpenoids with Potential Anti-Cancer Activity in Bran of Black Non-Glutinous Rice. *Nutrients* **7**, 1672-1687 (2015).
5. Tuckey, R.C. et al. Lumisterol is metabolized by CYP11A1: Discovery of a new pathway. *Int. J. Biochem. Cell Biol.* **55**, 24-34 (2014).
6. Fritzsching, K.J., Yang, Y., Schmidt-Rohr, K. & Hong, M. Practical use of chemical shift databases for protein solid-state NMR: 2D chemical shift maps and amino-acid assignment with secondary-structure information. *J. Biomol. NMR.* **56**, 155-167 (2013).
7. Ghosh, R., Xiao, Y., Kragelj, J. & Frederick, K.K. In-cell sensitivity-enhanced NMR of intact viable mammalian cells. *Journal of the American Chemical Society* **143**, 18454-18466 (2021).
8. Shim, J.H. et al. Antitumor Effect of Soluble b-1, 3-Glucan from Agrobacterium sp. R259 KCTC 1019. *J. Microbiol. Biotechnol.* **17**, 1513-1520 (2007).
9. Fairweather, J.K., Him, J.L.K., Heux, L., Driguez, H. & Bulone, V. Structural characterization by ^{13}C-NMR spectroscopy of products synthesized in vitro by polysaccharide synthases using ^{13}C-enriched glycosyl donors: application to a UDP-glucose:(1→ 3)-b-d-glucan synthase from blackberry (Rubus fruticosus). *Glycobiology* **14**, 775-781 (2004).
10. Saitô, H., Ohki, T. & Sasaki, T. A 13C-nuclear magnetic resonance study of polysaccharide gels. Molecular architecture in the gels consisting of fungal, branched (1→ 3)-b-D-glucans (lentinan and schizophyllan) as manifested by conformational changes induced by sodium hydroxide. *Carbohydr. Res.* **74**, 227-240 (1979).
11. Fernando, L.D. et al. Structural polymorphism of chitin and chitosan in fungal cell walls from solid-state NMR and principal component analysis. *Front. Mol. Biosci.*, 727053 (2021).
12. Lowman, D.W. et al. New Insights into the Structure of (1→3,1→6)-β-D-Glucan Side Chains in the Candida glabrata Cell Wall. *PLoS One* **6**, e27614 (2011).
13. Latge, J.P. et al. Chemical and immunological characterization of the extracellular galactomannan of Aspergillus fumigatus. *Infect. Immun.* **62**, 5424-5433 (1994).
14. Chakraborty, A. et al. A molecular vision of fungal cell wall organization by functional genomics and solid-state NMR. *Nat. Commun.* **12**, 6346 (2021).
15. Fontaine, T. et al. Galactosaminogalactan, a New Immunosuppressive Polysaccharide of Aspergillus fumigatus. *PLoS Pathog.* **7**, e1002372 (2011).
16. Kuraoka, T., Yamada, T., Takatsutsumi, Y., Ogawa, Y. & Kobayashi, H. Anomeric Proton and Carbon (H1-C1) NMR Chemical Shifts of Antigenic Mannans Obtained from Pathogenic Yeast Candida tropicalis. *Adv. Microbiol.* **11**, 296-301 (2021).
17. Kuraoka, T., Ishiyama, A., Oyamada, H., Ogawa, Y. & Kobayashi, H. Presence of O-glycosidically linked oligosaccharides in the cell wall mannan of Candida krusei purified with Benanomicin A. *FEBS Open Bio.* **9**, 129-136 (2018).
18. Archbald, P.J., Fenn, M.D. & Roy, A.B. 13C-N.M.R. studies of D-glucose and D-galactose monosulphates. *Carbohydr. Res.* **93**, 177-190 (1981).
